# Ethyl 2-[(carbamothioyl­amino)­imino]­propano­ate

**DOI:** 10.1107/S1600536811026237

**Published:** 2011-07-09

**Authors:** Charlane C. Corrêa, José Eugênio J.C. Graúdo, Luiz Fernando C. de Oliveira, Mauro V. de Almeida, Renata Diniz

**Affiliations:** a Núcleo de Espectroscopia e Estrutura Molecular (NEEM), Department of Chemistry, Federal University of Juiz de Fora, Minas Gerais 36036-900, Brazil; b Department of Chemistry, Federal University of Juiz de Fora, Minas Gerais 36036-900, Brazil

## Abstract

The title compound, C_6_H_11_N_3_O_2_S, consists of a roughly planar mol­ecule (r.m.s deviation from planarity = 0.077 Å for the non-H atoms) and has the S atom in an *anti* position to the imine N atom. This N atom is the acceptor of a strongly bent inter­nal N—H⋯N hydrogen bond donated by the amino group. In the crystal, mol­ecules are arranged in undulating layers parallel to (010). The mol­ecules are linked *via* inter­molecular amino–carboxyl N—H⋯O hydrogen bonds, forming chains parallel to [001]. The chains are cross-linked by N_carbazone_—H⋯S and C—H⋯S inter­actions, forming infinite sheets.

## Related literature

For the synthesis of thio­semicarbazones, see: Gupta & Narayana (1997 ▶); Li *et al.* (1998 ▶); Tarasconi *et al.* (2000 ▶); Holla *et al.* (2003 ▶); Shailendra *et al.* (2003 ▶). For the synthesis, crystal structures and applications of thio­semicarbazones, see: West *et al.* (1993 ▶); Casas *et al.* (2000 ▶); Beraldo (2004 ▶); Tenório *et al.* (2005 ▶). For graph-set notation, see: Etter *et al.* (1990 ▶).
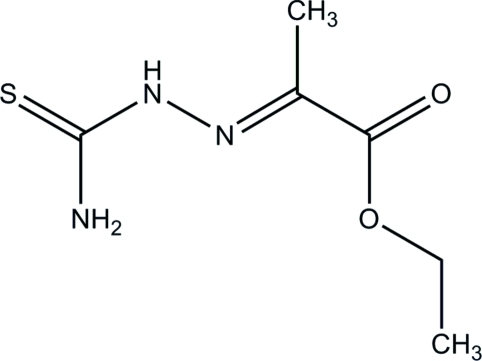


## Experimental

### 

#### Crystal data

C_6_H_11_N_3_O_2_S
*M* *_r_* = 189.24
Monoclinic, 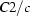

*a* = 16.682 (3) Å
*b* = 7.2558 (15) Å
*c* = 17.317 (4) Å
β = 116.63 (3)°
*V* = 1873.8 (7) Å^3^
*Z* = 8
Mo *K*α radiation
μ = 0.31 mm^−1^
*T* = 297 K
0.56 × 0.27 × 0.12 mm

#### Data collection

Bruker–Nonius KappaCCD diffractometer
15270 measured reflections
2133 independent reflections
1785 reflections with *I* > 2σ(*I*)
*R*_int_ = 0.021

#### Refinement

*R*[*F* ^2^ > 2σ(*F* ^2^)] = 0.033
*wR*(*F* ^2^) = 0.092
*S* = 1.08
2133 reflections
123 parameters
H atoms treated by a mixture of independent and constrained refinement
Δρ_max_ = 0.21 e Å^−3^
Δρ_min_ = −0.26 e Å^−3^

### 

Data collection: *COLLECT* (Hooft, 1999 ▶); cell refinement: *SCALEPACK* (Otwinowski & Minor, 1997 ▶); data reduction: *DENZO* (Otwinowski & Minor, 1997 ▶) and *SCALEPACK*; program(s) used to solve structure: *SHELXS97* (Sheldrick, 2008 ▶); program(s) used to refine structure: *SHELXL97* (Sheldrick, 2008 ▶); molecular graphics: *ORTEP-3 for Windows* (Farrugia, 1997 ▶) and *Mercury* (Macrae *et al.*, 2006 ▶); software used to prepare material for publication: *PLATON* (Spek, 2009 ▶).

## Supplementary Material

Crystal structure: contains datablock(s) global, I. DOI: 10.1107/S1600536811026237/qk2014sup1.cif

Structure factors: contains datablock(s) I. DOI: 10.1107/S1600536811026237/qk2014Isup2.hkl

Supplementary material file. DOI: 10.1107/S1600536811026237/qk2014Isup3.cml

Additional supplementary materials:  crystallographic information; 3D view; checkCIF report

## Figures and Tables

**Table 1:** Hydrogen-bond geometry (Å, °)

*D*—H⋯*A*	*D*—H	H⋯*A*	*D*⋯*A*	*D*—H⋯*A*
N1—H1*N*⋯N3	0.84 (2)	2.24 (2)	2.610 (2)	107 (2)
N1—H2*N*⋯O2^i^	0.88 (3)	2.08 (3)	2.954 (2)	172 (2)
N2—H3*N*⋯S1^ii^	0.85 (2)	2.78 (2)	3.623 (2)	172 (2)
C3—H3*C*⋯S1^ii^	0.96	2.82	3.611 (2)	141

Symmetry codes: (i) 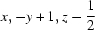; (ii) 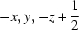.

**Fig. 1.**
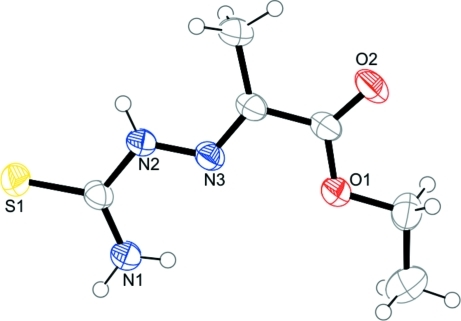
The molecular structure of ethyl pyruvate thiosemicarbazone showing 50% displacement ellipsoids.

**Fig. 2.**
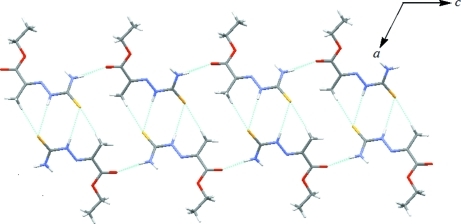
View of a ribbon of hydrogen bonded molecules in the crystal structure of ethyl pyruvate thiosemicarbazone.

## supplementary crystallographic information

### Comment

Thiosemicarbazones are a class of substances known for their biological and chemical properties, such as antiviral, antibacterial, antiprotozoal and antitumor activity (West *et al.*, 1993). The enzyme ribonucleoside diphosphate reductase (RDR) (Beraldo, 2004) is an object of attack by thiosemicarbazones, which relates to their tumor control properties. One background for the biological activity of thiosemicarbazones is certainly their ability to form chelates with transition metal ions. The synthesis of thiosemicarbazones is described in several works in the literature (Gupta & Narayana, 1997; Li *et al.*, 1998; Tarasconi *et al.*, 2000; Holla *et al.*, 2003; Shailendra *et al.*, 2003). In context with potential biological activity the crystal structure determination of the title compound, ethyl pyruvate thiosemicarbazone (scheme 1), was of interest. The compound may also be interesting as ligand in coordination chemistry.

Figure 1 shows the *ORTEP* representation of the asymmetric unit of the title compound. The compound features a fairly planar molecule with a r.m.s deviation from planarity 0.077 Å for the non-hydrogen atoms. The sulfur atom is in *anti* position to the imine nitrogen N3. The bond lengths in the N—C(S)—N fragment indicate delocalization of the π electrons due to the fact that the C—N and C—S bonds are shorter than tipycal single bonds (around 1.47 and 1.73 Å, respectively) and bigger than corresponding double bonds (around 1.29 and 1.55 Å, respectively) (Casas *et al.*, 2000; Tenório *et al.*, 2005). The molecule is stabilized by the strongly bent intramolecular hydrogen N1—H1n···N3, N1···N3 = 2.610 (2) Å (Table 1).

In the crystal lattice the molecules are arranged in undulating layers parallel to (010). Via the intermolecular hydrogen bond N1—H2n···O2*^i^* the molecules are linked to form continuous chains parallel to [001], as visualized in Figure 2. The graph-set representation for this arrangement is N = C(8), (Etter *et al.*, 1990). Each two of these chains are mutually crosslinked by the weak interactions N2—H3n···S1*^ii^*, N2···S1*^ii^* = 3.623 (2) Å, and C3—H3c···S1*^ii^*, C3···S1*^ii^* = 3.611 (2) Å, to form infinite ribbons along the *c* axis, see Table 1 and Fig. 2.

### Experimental

For preparation of the title compound, 0.188 g of ethyl pyruvate (1.62 mmol) was added to 15 ml of a water-methanol solution (1:2) of thiosemicarbazide hydrochloride (1.48 g, 1.62 mmol) and the mixture was heated at 80 °C for 3 h. After few days, colorless crystals formed at room temperature and were isolated. M.p.: 145 °C. Elemental analysis gave the following results: Calcd. for C_6_H_11_O_2_N_3_S: C 38.08, H 5.86, N 22.21%; found: C 39.69; H 6.62; N 22.93%.

IR spectral data were obtained with a Bomem MB-102 spectrometer fitted with a CsI beam splitter, using KBr disks and a spectral resolution of 4 cm^-1^. The main absorption bands are (cm^-1^): 3442–3204 (νNH); 1709 (νCO); 1600 (νNH + νCN + νCC); 1498 (νCO_asym_); 1370 (νCN); 1173 (νCO_sym_); 1119, 1024 (νCS) ; 1105 (νCOC).

### Refinement

C-bound H atoms were included in the riding model approximation with C—H = 0.96 Å and *U*_iso_(H) = 1.5×*U*_equ_(C) for CH_3_, and 0.97 Å *U*_iso_(H) = 1.2×*U*_equ_(C) for CH_2_. H atoms of nitrogen atoms were located from an electron density map and were refined unrestrained in *x,y,z*, and *U*_iso_.

### Crystal data

**Table:**

C_6_H_11_N_3_O_2_S	*F*(000) = 800
*M**_r_* = 189.24	*D*_x_ = 1.342 Mg m^−^^3^
Monoclinic, *C*2/*c*	Melting point: 418 K
Hall symbol: -C 2yc	Mo *K*α radiation, λ = 0.71073 Å
*a* = 16.682 (3) Å	Cell parameters from 106 reflections
*b* = 7.2558 (15) Å	θ = 4.7–22.6°
*c* = 17.317 (4) Å	µ = 0.31 mm^−^^1^
β = 116.63 (3)°	*T* = 297 K
*V* = 1873.8 (7) Å^3^	Prism, colourless
*Z* = 8	0.56 × 0.27 × 0.12 mm

### Data collection

**Table:**

Bruker–Nonius KappaCCD diffractometer	1785 reflections with *I* > 2σ(*I*)
Radiation source: fine-focus sealed tube	*R*_int_ = 0.021
horizonally mounted graphite crystal	θ_max_ = 27.5°, θ_min_ = 5.3°
Detector resolution: 9 pixels mm^-1^	*h* = −21→21
ω and φ scans	*k* = −9→9
15270 measured reflections	*l* = −22→22
2133 independent reflections	

### Refinement

**Table:**

Refinement on *F*^2^	0 restraints
Least-squares matrix: full	Secondary atom site location: difference Fourier map
*R*[*F*^2^ > 2σ(*F*^2^)] = 0.033	H atoms treated by a mixture of independent and constrained refinement
*wR*(*F*^2^) = 0.092	*w* = 1/[σ^2^(*F*_o_^2^) + (0.0423*P*)^2^ + 1.1077*P*] where *P* = (*F*_o_^2^ + 2*F*_c_^2^)/3
*S* = 1.08	(Δ/σ)_max_ < 0.001
2133 reflections	Δρ_max_ = 0.21 e Å^−^^3^
123 parameters	Δρ_min_ = −0.26 e Å^−^^3^

### Special details

**Table:**

Geometry. All e.s.d.'s (except the e.s.d. in the dihedral angle between two l.s. planes) are estimated using the full covariance matrix. The cell e.s.d.'s are taken into account individually in the estimation of e.s.d.'s in distances, angles and torsion angles; correlations between e.s.d.'s in cell parameters are only used when they are defined by crystal symmetry. An approximate (isotropic) treatment of cell e.s.d.'s is used for estimating e.s.d.'s involving l.s. planes.
Refinement. Refinement of *F*^2^ against ALL reflections. The weighted *R*-factor *wR* and goodness of fit *S* are based on *F*^2^, conventional *R*-factors *R* are based on *F*, with *F* set to zero for negative *F*^2^. The threshold expression of *F*^2^ > σ(*F*^2^) is used only for calculating *R*-factors(gt) *etc*. and is not relevant to the choice of reflections for refinement. *R*-factors based on *F*^2^ are statistically about twice as large as those based on *F*, and *R*- factors based on ALL data will be even larger.

### Fractional atomic coordinates and isotropic or equivalent isotropic displacement parameters (Å^2^)

**Table:**

	*x*	*y*	*z*	*U*_iso_*/*U*_eq_	
S1	0.07816 (2)	0.66899 (7)	0.18273 (2)	0.05007 (15)	
O1	0.36286 (7)	0.42826 (16)	0.55879 (6)	0.0453 (3)	
O2	0.29362 (8)	0.4511 (2)	0.64308 (7)	0.0599 (4)	
N3	0.21829 (8)	0.52146 (17)	0.42323 (7)	0.0363 (3)	
N2	0.14791 (8)	0.57872 (18)	0.34827 (7)	0.0394 (3)	
N1	0.23628 (9)	0.5179 (2)	0.28107 (9)	0.0541 (4)	
C2	0.21056 (9)	0.5244 (2)	0.49384 (9)	0.0365 (3)	
C1	0.15936 (9)	0.5823 (2)	0.27447 (9)	0.0368 (3)	
C4	0.29221 (9)	0.4639 (2)	0.57299 (9)	0.0378 (3)	
C3	0.13145 (10)	0.5866 (3)	0.50629 (11)	0.0507 (4)	
H3A	0.1298	0.7189	0.5069	0.076*	
H3B	0.1365	0.5398	0.5601	0.076*	
H3C	0.0773	0.5410	0.4598	0.076*	
C6	0.51684 (12)	0.3513 (3)	0.60556 (14)	0.0644 (5)	
H6A	0.4977	0.2588	0.5613	0.097*	
H6B	0.5713	0.3122	0.6537	0.097*	
H6C	0.5272	0.4650	0.5831	0.097*	
C5	0.44536 (10)	0.3796 (3)	0.63469 (11)	0.0503 (4)	
H5A	0.4368	0.2677	0.6607	0.060*	
H5B	0.4626	0.4777	0.6772	0.060*	
H1N	0.2740 (14)	0.476 (3)	0.3288 (14)	0.061 (6)*	
H3N	0.0969 (12)	0.612 (2)	0.3430 (11)	0.045 (4)*	
H2N	0.2477 (13)	0.525 (3)	0.2361 (14)	0.060 (5)*	

### Atomic displacement parameters (Å^2^)

**Table:**

	*U*^11^	*U*^22^	*U*^33^	*U*^12^	*U*^13^	*U*^23^
S1	0.0402 (2)	0.0741 (3)	0.0365 (2)	0.00740 (18)	0.01775 (16)	0.01065 (17)
O1	0.0387 (5)	0.0663 (7)	0.0337 (5)	0.0055 (5)	0.0186 (4)	0.0026 (5)
O2	0.0558 (7)	0.0988 (10)	0.0322 (5)	0.0012 (6)	0.0260 (5)	−0.0006 (6)
N3	0.0358 (6)	0.0444 (6)	0.0318 (5)	−0.0029 (5)	0.0179 (5)	−0.0016 (5)
N2	0.0344 (6)	0.0552 (8)	0.0339 (6)	0.0028 (5)	0.0200 (5)	0.0027 (5)
N1	0.0421 (7)	0.0910 (12)	0.0369 (7)	0.0153 (7)	0.0246 (6)	0.0118 (7)
C2	0.0385 (7)	0.0425 (7)	0.0343 (6)	−0.0052 (6)	0.0213 (6)	−0.0043 (6)
C1	0.0352 (6)	0.0456 (8)	0.0333 (6)	−0.0042 (6)	0.0185 (5)	−0.0004 (6)
C4	0.0415 (7)	0.0440 (8)	0.0334 (7)	−0.0053 (6)	0.0216 (6)	−0.0056 (6)
C3	0.0431 (8)	0.0733 (11)	0.0454 (8)	0.0025 (8)	0.0284 (7)	−0.0012 (8)
C6	0.0447 (9)	0.0665 (12)	0.0798 (13)	0.0050 (8)	0.0259 (9)	−0.0007 (10)
C5	0.0430 (8)	0.0574 (10)	0.0432 (8)	0.0009 (7)	0.0128 (7)	0.0070 (7)

### Geometric parameters (Å, °)

**Table:**

S1—C1	1.6808 (16)	N1—H1N	0.84 (2)
O1—C4	1.3325 (17)	N1—H2N	0.88 (2)
O1—C5	1.4577 (19)	N2—H3N	0.85 (2)
O2—C4	1.2069 (17)	C3—H3A	0.9600
N3—C2	1.2860 (17)	C3—H3B	0.9600
N3—N2	1.3675 (17)	C3—H3C	0.9600
N2—C1	1.3745 (17)	C5—H5A	0.9700
N1—C1	1.3217 (19)	C5—H5B	0.9700
C2—C3	1.4989 (19)	C6—H6A	0.9600
C2—C4	1.501 (2)	C6—H6B	0.9600
C6—C5	1.503 (2)	C6—H6C	0.9600
			
C4—O1—C5	115.86 (11)	C2—C3—H3A	109.00
C2—N3—N2	119.23 (12)	C2—C3—H3B	109.00
N3—N2—C1	118.06 (11)	C2—C3—H3C	109.00
N3—C2—C3	127.72 (14)	H3A—C3—H3B	109.00
N3—C2—C4	115.25 (12)	H3A—C3—H3C	109.00
C3—C2—C4	117.00 (12)	H3B—C3—H3C	109.00
N1—C1—N2	116.55 (13)	O1—C5—H5A	110.00
N1—C1—S1	123.64 (11)	O1—C5—H5B	110.00
N2—C1—S1	119.80 (11)	C6—C5—H5A	110.00
O2—C4—O1	123.40 (14)	C6—C5—H5B	110.00
O2—C4—C2	122.77 (13)	H5A—C5—H5B	108.00
O1—C4—C2	113.83 (11)	C5—C6—H6A	109.00
O1—C5—C6	107.52 (14)	C5—C6—H6B	109.00
C1—N1—H1N	118.9 (17)	C5—C6—H6C	109.00
C1—N1—H2N	119.2 (15)	H6A—C6—H6B	109.00
H1N—N1—H2N	122 (2)	H6A—C6—H6C	109.00
C1—N2—H3N	116.4 (12)	H6B—C6—H6C	109.00
N3—N2—H3N	125.5 (12)		
			
C5—O1—C4—O2	−2.7 (2)	N2—N3—C2—C3	−0.6 (2)
C5—O1—C4—C2	176.48 (14)	N2—N3—C2—C4	−178.40 (13)
C4—O1—C5—C6	−178.14 (15)	N3—C2—C4—O1	4.43 (19)
C1—N2—N3—C2	177.04 (14)	N3—C2—C4—O2	−176.40 (15)
N3—N2—C1—S1	−174.66 (11)	C3—C2—C4—O1	−173.59 (15)
N3—N2—C1—N1	4.4 (2)	C3—C2—C4—O2	5.6 (2)

### Hydrogen-bond geometry (Å, °)

**Table:**

*D*—H···*A*	*D*—H	H···*A*	*D*···*A*	*D*—H···*A*
N1—H1N···N3	0.84 (2)	2.24 (2)	2.610 (2)	107 (2)
N1—H2N···O2^i^	0.88 (3)	2.08 (3)	2.954 (2)	172 (2)
N2—H3N···S1^ii^	0.85 (2)	2.78 (2)	3.623 (2)	172 (2)
C3—H3C···S1^ii^	0.96	2.82	3.611 (2)	141

Symmetry codes: (i) *x*, −*y*+1, *z*−1/2; (ii) −*x*, *y*, −*z*+1/2.
